# TAGCNA: A Method to Identify Significant Consensus Events of Copy Number Alterations in Cancer

**DOI:** 10.1371/journal.pone.0041082

**Published:** 2012-07-18

**Authors:** Xiguo Yuan, Junying Zhang, Liying Yang, Shengli Zhang, Baodi Chen, Yaojun Geng, Yue Wang

**Affiliations:** 1 School of Computer Science and Technology, Xidian University, Xi'an, People’s Republic of China; 2 Bradley Department of Electrical and Computer Engineering, Virginia Polytechnic Institute and State University, Arlington, Virginia, United States of America; 3 Department of Mathematics, Xidian University, Xi'an, People’s Republic of China; Northwestern University Feinberg School of Medicine, United States of America

## Abstract

Somatic copy number alteration (CNA) is a common phenomenon in cancer genome. Distinguishing significant consensus events (SCEs) from random background CNAs in a set of subjects has been proven to be a valuable tool to study cancer. In order to identify SCEs with an acceptable type I error rate, better computational approaches should be developed based on reasonable statistics and null distributions. In this article, we propose a new approach named TAGCNA for identifying SCEs in somatic CNAs that may encompass cancer driver genes. TAGCNA employs a peel-off permutation scheme to generate a reasonable null distribution based on a prior step of selecting tag CNA markers from the genome being considered. We demonstrate the statistical power of TAGCNA on simulated ground truth data, and validate its applicability using two publicly available cancer datasets: lung and prostate adenocarcinoma. TAGCNA identifies SCEs that are known to be involved with proto-oncogenes (*e.g.* EGFR, CDK4) and tumor suppressor genes (*e.g.* CDKN2A, CDKN2B), and provides many additional SCEs with potential biological relevance in these data. TAGCNA can be used to analyze the significance of CNAs in various cancers. It is implemented in R and is freely available at http://tagcna.sourceforge.net/.

## Introduction

Somatic copy number alterations (CNAs) are distributed throughout the genome in almost all human cancers [1]. One of the systematical efforts in exploring the effect of CNAs on cancer development is to distinguish significant consensus events (SCEs) that represent “driver mutations” from random background CNAs that represent “passenger mutations” [2], [3]. Extremely high resolution array technologies and large collection of cancer subjects further a comprehensive understanding of the mutational events in such a program [1], [3], [4]. This meanwhile leads to a critical requirement of computational approaches for identifying significance aberrations that are shared by multiple subjects.

Currently, many statistical approaches have been developed. STAC (Significance Testing for Aberrant Copy number) [5] tests CNAs separately for amplifications and deletions, and it requires binary input data matrices, in which ‘one’ represents amplification (or deletion) and “zero” represents normal status. This method utilizes two complementary statistics: frequency and footprint, to measure each marker under the null hypothesis that the observed CNA regions are equally placed anywhere across the genome being analyzed. Specifically, the “frequency” statistic is used to reflect the commonness of an aberration across samples and the “footprint” statistic is used to reflect the tight alignment of an aberrant region across samples. Furthermore, “footprint” takes into account the correlations among aberrations and the lengths of CNA regions. However, both of the statistics have not incorporated the amplitude of aberrations, so that some important information may be missed, since high-level amplifications and deletions may lead to different biological implications compared to low-level aberrations [6]. Similar to STAC, GISTIC (Genomic Identification of Significant Targets In Cancer) [3] also analyzes amplifications and deletions separately, but it requires input data with segmented signals. This method designs a G-score by incorporating both the frequency and amplitude of aberrations, and assigns the G-score to each marker for assessing significance based on a semi-exactly approximated null distribution. The null distribution is established by assuming that CNA markers are independent. Accordingly, the joint effects between adjacent markers are ignored in CNA detections [7]. To improve the detection power, an extension of GISTIC, GISTIC2.0 [8], is proposed, which considers the distinction of the background frequency between focal CNAs and broad CNAs and scores each marker proportional to its amplitude. Another similar method is the DiNAMIC (Discovering Copy Number Aberrations Manifested In Cancer) [9], which defines a summary statistic for each marker and designs a new framework for the significance assessment. It employs a cyclic permutation scheme to generate null distribution, in which the structural information of the original copy number data is maintained. DiNAMIC further adopts a 'peel-off' algorithm to detect less-frequent markers. In general, the feature that the above methods share is their two-stage approach, i.e. they need a prior step of discretizing the CNA signals using individual-sample analysis methods [10], [11]. To avoid dependence on individual-sample analysis, many authors propose one-stage computational approach. For instance, KC-SMART (Kernel Convolution: a Statistical Method for Aberrant Region deTection) [12] directly analyzes raw intensity ratio data (i.e. the data without discretization in individual samples) to identify SCEs using a new statistic: Kernel Smoothed Estimate (KSE), which takes into account the signal strength of neighboring markers; and CMDS (Correlation Matrix Diagonal Segmentation) [13] scores each marker based on its correlations with the surrounding sites in the raw intensity ratios. Many other approaches are discussed by Shah [14] and Rueda *et al.* [7].

Within the existing approaches, three common and important components are summarized as follows: (1) data platform, i.e. raw intensity ratio data or discretized data (corresponding to one-stage or two-stage approach), for detecting SCEs; (2) statistic associated with genomic units (*e.g.* markers or genes); and (3) null distribution for testing the statistic. However, one surprisingly difficult question here is how to make a consistency among the three components, taking into account CNA structures and statistical significance. So far, there are no definite solutions to this question. One-stage methods may lead to a large bias signal to the statistics [15], in which the null distribution is not exactly consistent with the purpose of identifying SCEs from random background CNAs, *e.g.* the null hypothesis underlying CMDS is that there is no CNA. In this case, SCE detection power may be greatly affected by the occurrence probability of random CNAs. Two-stage methods often utilize the defined CNAs (gains or losses) to generate null distribution through permutations. However, many of them adopt marker-based scoring but region-based permutation schemes, such as STAC and DiNAMIC methods. GISTIC program makes out a reasonable consistency among the three components, but it does not consider the correlations among markers. This might make the statistical significance conservative in multiple testing [16], and may not be biologically relevant [7]. Overall, most existing methods in either one-stage or two-stage frameworks quantify CNAs and test the significance based on individual markers, which are usually related with each other. This may lead to a decreased power in detecting CNA regions especially for those less-extreme regions [7]. Furthermore, they usually generate null distributions based on a mixture of SCEs (false null hypotheses) and random background CNAs (true null hypotheses). This is theoretically deviated from the true null distribution in statistical hypothesis testing, decreasing the meaning of significance assessment.

With these considerations, in this article we propose a new approach, TAGCNA, for identifying SCEs based on continuous segmented signal ratios. The approach is composed of two steps. First, select tag CNA markers from the genome being analyzed, and then produce a new data matrix consisting of tag markers, each of which is scored by incorporating both frequency and amplitude of CNA; and second, based on the data matrix, create a null distribution using a peel-off permutation scheme. The primary features of the approach include: (1) both scoring and permutation are performed based on tag marker-level, considering the correlations among adjacent markers; (2) the mean of the null distribution moves left due to the peel-off procedure on tag markers, converging to that of the truth null distribution. TAGCNA can be used to analyze data from individual chromosomes as well as data derived from genome-wide studies. We test its statistical power on extensive simulated ground truth data, and then apply it to two real datasets of lung and prostate cancers. TAGCNA successfully identifies SCEs associated with known cancer driver genes, and provides many additional SCEs with potential biological relevance.

## Materials and Methods

### Data Format

Original data is preprocessed through individual-sample analysis methods such as CBS [10], [17], and is stored in matrix *X* (*N*×*L*), where each row represents a subject and each column represents a marker. TAGCNA starts work from this point. It adopts thresholds (*θ* ^amp^ and *θ* ^del^) to define amplifications and deletions in *X*, and separates *X* into two matrices *X* ^amp^ (*N*×*L*) and *X* ^del^ (*N*×*L*). TAGCNA analyzes amplification and deletion separately since they are generally regarded as playing distinct roles in cancer development.

In matrix *X* ^amp^ (or *X* ^del^), aberration is represented with a log_2_-ratio, and no aberration is represented with a zero. Below we describe the TAGCNA principle to test significance of CNAs either in the analysis of amplification or deletion data matrix.

### Selecting Tag CNA Markers

Somatic CNA is a structural variation in the human genome, thus the probes in the genome are inherently correlated even if the CNAs are random background events. It is desirable to maintain this correlation and to maximize the independence between test statistics in the analysis of CNAs. These considerations led us to design TAGCNA to test CNAs by partitioning the genome into small correlation blocks and selecting tag markers in different blocks, which are assumed independent. Scoring and permutation procedures of TAGCNA are then performed on the tag markers.

**Figure 1:**
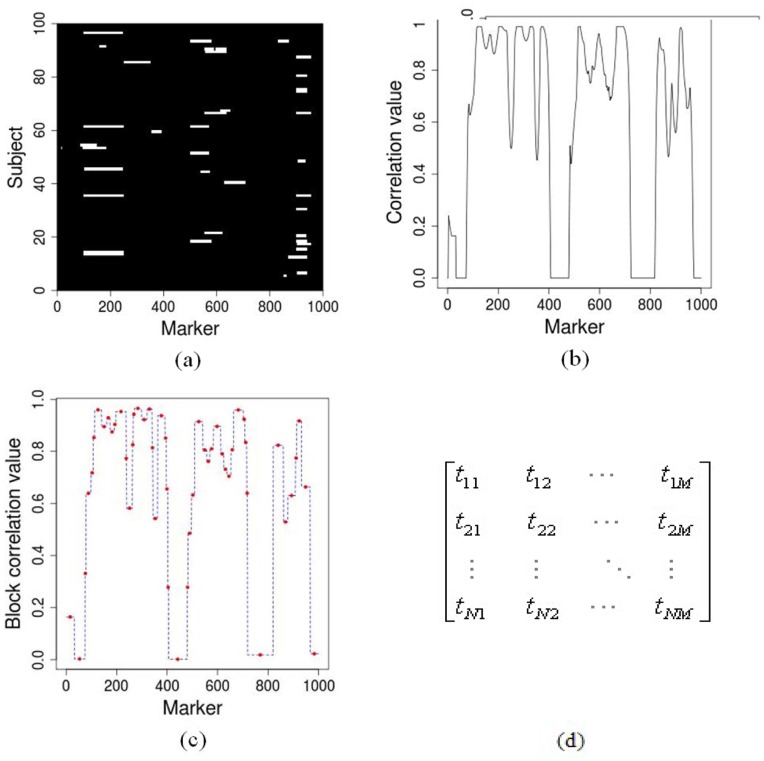
An example of tag marker selection procedure, (a) → (b) → (c) → (d). (a) A matrix profile of 100 subjects and 1000 markers; the white colored positions indicate copy number alterations. (b) The correlation value for each marker, which is the average coefficient among its surrounding markers. (c) Block correlation value resulted from the partition of the genome based on (b). (d) A new data matrix consisting of tag CNA markers (here *N*  = 100, *M*  = 50); each tag marker is selected from each block in (c), where the red dots are the middle of the blocks, representing tag markers.

CNA correlation block partition is carried out based on a set of subjects (Figure 1). The first step is to calculate correlation coefficients between adjacent markers via Pearson correlation formula [13]:
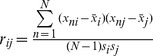
(1)where *r_ij_* is the correlation coefficient between markers *i* and *j*; *N* is the number of samples; *x_ni_* is log_2_-ratio of subject *n* at marker *i*; 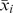, 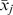, 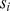 and 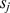 are log_2_-ratio means and standard deviations of markers *i* and *j* across all subjects. Then we obtain a correlation value for each marker *k* by averaging coefficients among its surrounding markers by Equation (2) [13]:
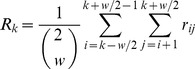
(2)where *w* is a pre-specified window size around marker *k*. Figure 1 (b) shows the correlation value for the 1000 markers in the exampled population. To utilize the spatial coherence among adjacent markers, we assume that the correlation values in the nearby markers are at the same level and employ CBS algorithm [10] to partition the whole genome into blocks where correlation values change between contiguous blocks (Figure 1 (c)). In each block, one tag marker is selected from its middle site. Thus, the total number of tag markers is the number of blocks resulted from the partition of the genome. A new data matrix T (*N×M*) is then produced based on the tag markers (Figure 1 (d)), where *M* is the number of tag markers.

### Peel-off Permutation and Assessing Statistical Significance

Based on the data matrix *T*, TAGCNA performs peel-off permutation [3], [9] to generate null distribution under the hypothesis that there is no SCEs, i.e. that all tag markers in *T* are passengers, and then assesses the statistical significance of the observed tag markers. To mirror this, TAGCNA scores each tag marker *m* by incorporating frequency and amplitude of CNA [3]:
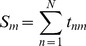
(3)where *t_nm_* is log_2_-ratio of subject *n* at tag marker *m* in matrix *T*. Note that the significance of the tag marker is supposed to represent the significance of the corresponding genome block.

**Figure 2:**
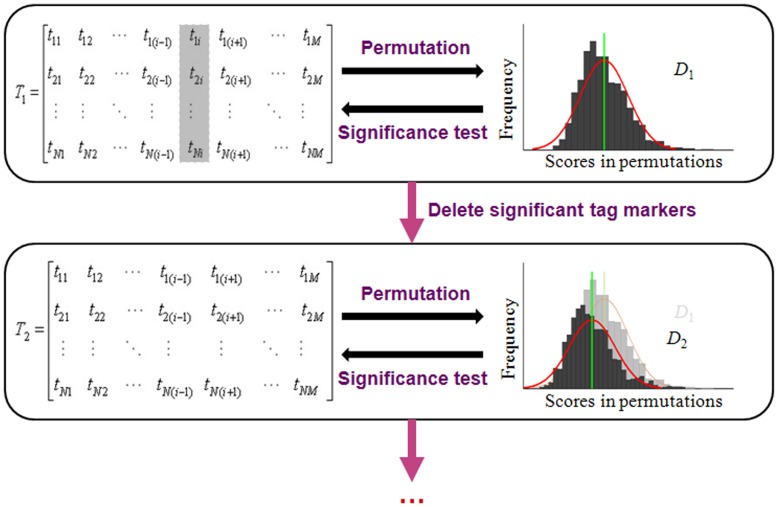
Procedure of peel-off permutation and significance test. It starts from the tag marker data matrix *T* (*N* ×*M*), and generates null distribution *D*_1_ through permutations on the data. Based on *D*_1_, significance level is assigned to each tag marker. If the significance level is less than a cutoff (*e.g.* 0.05), the corresponding markers (*e.g.* the *i*-th tag marker) will be removed from the matrix in the next iteration of permutation and significance test. This procedure is continuing until achieving a null distribution *D_H_*, based on which there are no additional tag markers are identified significant. In this procedure, the mean of the null distribution moves left gradually, *e.g.* in the second iteration, *D*_2_ moves left when compared with *D*_1_.

We now describe the procedure of peel-off permutation and significance test in detail, which is also illustrated in Figure 2. At the beginning, a null distribution *D*_1_ is estimated using permutation on the matrix *T*_1_ (*T*_1_ =  *T*). Based on *D*_1_, each tag marker is assigned a p-value. This algorithm can be decomposed into the following steps:

1. In each subject, perform a permutation of the tag markers, i.e. randomly place the tag markers in the tag locations of the genome.
2. In the permuted dataset *δ*(*T*_1_), calculate the score over tag marker *m*, denoted by *S_m_*(*δ*(*T*_1_)), *m*  = 1, 2, …, *M*.
3. Repeat steps (1) and (2) *E* times, i.e. perform *E* permutations of the dataset, and thus obtain *E* permuted datasets *δ* ^1^(*T*_1_), *δ* ^2^(*T*_1_), …, *δ^E^*(*T*_1_), and the corresponding scores *S_m_*(*δ* ^1^(*T*_1_)), *S_m_* (*δ* ^2^(*T*_1_) ), …, *S_m_* (*δ^E^*(*T*_1_) ).
4. Let *D*_1_ be the distribution of max*_m_ S_m_*(*δ*(*T*_1_)) over all the *E* permutations, and define the p-value for tag marker *m*_0_ (*m*_0_∈{1…*M* }) by the extreme right-hand probability [5], [9]:
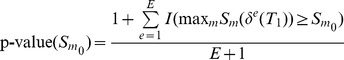
(4)where I (·) is the indicator function.

Subsequently, TAGCNA scans the p-values across all the tag markers. If any one or more of the p-values are less than a significance cutoff (*e.g.* 0.05), the corresponding tag markers will deleted (Figure 2). Then a new data matrix *T*_2_ is produced without incorporating the significant tag markers. Based on *T*_2_, a null distribution *D*_2_ can be created via the above four steps and the significance level of the remainder tag markers can be assessed.

The procedure is continuing until achieving a null distribution *D_H_*, based on which no additional tag markers can be identified significant. During the procedure, a sequence of data matrices *T*_1_, *T*_2_, …, *T_H_* and a sequence of null distributions *D*_1_, *D*_2_, …, *D_H_* are obtained. We observe that the number of columns in the data matrices are decreasing and the means of the null distributions are moving left gradually along with the sequence. This implies that *T_H_* might not include highly-extreme tag markers and the proportion of true null hypotheses is greatly increased, so the resulted null distribution *D_H_* might be extremely close to the truth null distribution. Finally, based on *D_H_*, TAGCNA assesses the significance levels of all the observed tag markers again. This might improve the power for identifying less-extreme SCEs and also correct the p-values in terms of statistical significance.

## Results

### Simulation Studies

Real datasets rarely have absolutely confirmed ground truth SCEs, so it is difficult to assess the performance of statistical methods on real data. In this section, we design simulation studies to test the statistical power of our approach. The simulation model proposed by Willenbrock and Fridlyand [18] is modified to generate CNA datasets under various parameter settings. In each setting, we simulate 100 subjects each with 10000 markers. Log_2_-ratio for each subject is generated by mixing normal and tumor cells. The proportion of normal cell for a particular subject is drawn from a uniform distribution between 0.3 and 0.7. Gaussian noise of mean zero and varying variance is added to each subject. Here we consider three levels of the variance in the Gaussian noise distribution, i.e. its standard deviation (SD) (σ) is drawn uniformly from [0.1, 0.2], [0.2, 0.4], or [0.4, 0.6] [18] in the simulation of each subject. To further make the simulation more realistic, we add two non-SCE regions with length ranging from 50 to 500 to each subject. The positions of the non-SCE regions are randomly selected in the stretch of the simulated genome, and the log_2_-ratios of the regions are generated uniformly between 0.585 (copies 3) and 1.322 (copies 5). Three ground truth SCEs are embedded in the simulated datasets. The log_2_-ratios and lengths of them are specified as Ratio  =  {0.585, 1, 1.322} and L  =  {200, 100, 50}, respectively. The frequency of all the three SCEs across subjects is denoted as *f*. Two frequency levels, 0.15 and 0.20, are considered for simulating various genome datasets.

**Figure 3:**
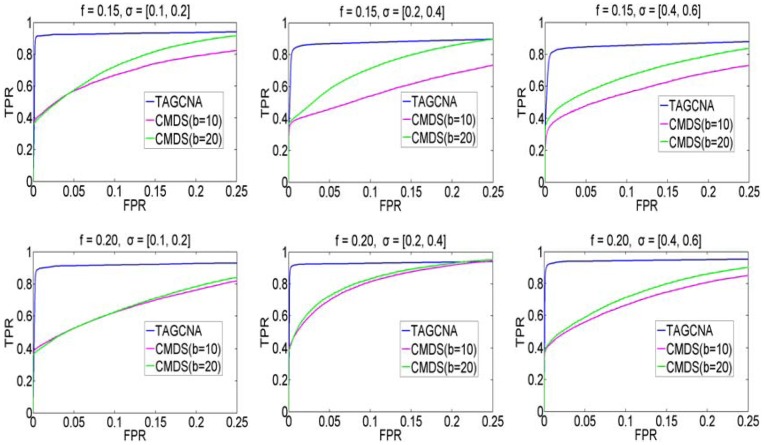
Performance comparison between CMDS and TAGCNA based on ROC curves. TPR and FPR are averaged over 100 simulated replications in each parameter setting. We use two options (i.e. b = 10 and b = 20) for the CMDS method in the data analysis.

We implement TAGCNA on the simulated datasets by setting the parameters *θ^a^* ^mp^ and *θ* ^del^ to 0.1 and −0.1, as well as *w* to 20, and compare its performance against CMDS [13] based on ROC curves, which are shown in Figure 3. Each ROC curve is plotted for one simulation parameter setting, in which the TPR (true positive rate) versus FPR (false positive rate) is calculated at different significance levels and is then averaged over 100 simulated replications. From Figure 3 we can note that in most cases, TAGCNA is more powerful than CMDS in terms of larger areas under the ROC curves. Therefore, TAGCNA is a valuable tool in identifying SCEs from background CNAs.

Additionally, to study the behaviour of TAGCNA under the true null hypothesis that there are no SCEs, we adopt the algorithm introduced by Walter et al. [9] to simulate null CNA datasets and perform TAGCNA on these data. Again, three levels of Gaussian noise are considered in the simulation scheme in an effort to show the robust behaviour of TAGCNA. The results of these experiments are shown in Table 1. In each case, the type I error rate resulted by TAGCNA is calculated according to the following steps:

1. Simulate 600 replications using the simulation algorithm with default parameter setting in Walter et al's work [9].
2. For each data replication, implement TAGCNA based on 1000 permutations, and determine if there are any CNAs are significant at p-value <0.05.
3. Calculate the number of replications in which there exist significant CNAs, and define the type I error rate as the proportion of these replications in the 600 replications.

The values of the type I error rate listed in Table 1 are very close to 0.05, indicating that TAGCNA is slightly conservative and the permutation procedure on tag CNA markers is relatively reasonable.

**Table 1:** Type I error rate for null CNA datasets analyzed by TAGCNA.

Null simulation*	Type I error rate
[0.1, 0.2]	0.0433
[0.2, 0.4]	0.0483
[0.4, 0.6]	0.0450

* The interval of the standard deviation (SD) of the Gaussian noise added to the simulated datasets, i.e. the SD of the Gaussian noise for each CNA marker is drawn uniformly from the interval.

### Application to Real Datasets

We applied TAGCNA to two publicly available cancer datasets. The first consists of 371 lung adenocarcinoma subjects, each of which includes 216,327 markers. This dataset is obtained from the TSP (Tumor Sequencing Project) project and is available at http://www.broadinstitute.org/cancer/pub/tsp/ [19]. The second set is generated from 82 prostate adenocarcinoma subjects in TCGA (The Cancer Genome Atlas) project, each subject was profiled using SNP6.0 in 1,868,857 markers, and the data is available at http://cancergenome.nih.gov/. Original CNA data are segmented via individual-sample analysis and are transformed into the input format to TAGCNA as described in the software package document. TAGCNA is implemented in each chromosome for analyzing amplification and deletion separately. We set the log_2_-ratio thresholds *θ* ^amp^ and *θ* ^del^ to 0.848 (3.6 copies) and −0.737 (1.2 copies), which is the setting of the GISTIC method in analyzing cancer genomes [19], as well as parameter *w* to 20, and perform 1000 random permutations to assess the significance of tag markers. Tag markers with p-values less than 0.05 are considered significant, and accordingly the relevant genome blocks are considered as SCEs.

#### Result on the lung adenocarcinoma dataset

**Figure 4:**
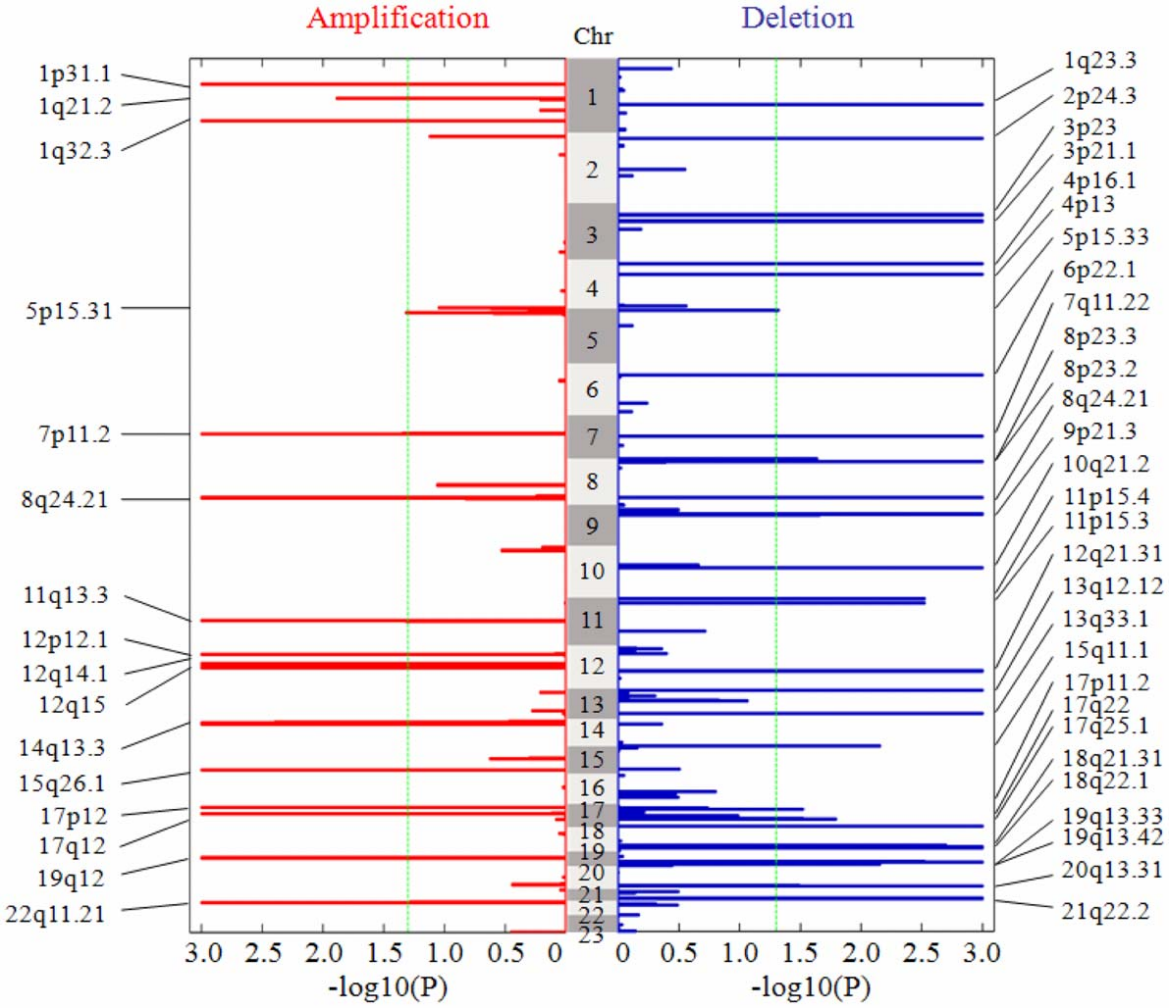
The landscape of statistical significance levels of the genome in the 371 lung adenocarcinoma subjects. −log10 (p-values) are given for amplification and deletion regions respectively. The dashed green line is placed at 1.3 (corresponding p-value of 0.05) as a cutoff for calling significant consensus events. Chromosome 23 indicates the sex chromosome.

Figure 4 shows the significance landscape of the whole genome resulted from the analysis of the lung adenocarcinoma dataset. TAGCNA identifies a total of 16 amplifications and 29 deletions in different chromosomes as listed in the both sides of Figure 4. The genes covered by these SCEs are given in Table S1. Many known cancer driver genes are included in the result. For instance, EGFR (epidermal growth factor receptor) is an oncogene contained in 7p11.2 (p-value <0.001). Its amplifications can result in over expression and uncontrolled cell division, which is a predisposition for cancer [20]. The maximum inferred copy number at 7p11.2 is 9.1, and there are 11 (3%) subjects with copy number above threshold 3.6 at the region and 50 (13.5%) subjects above threshold 2.5.

**Figure 5:**
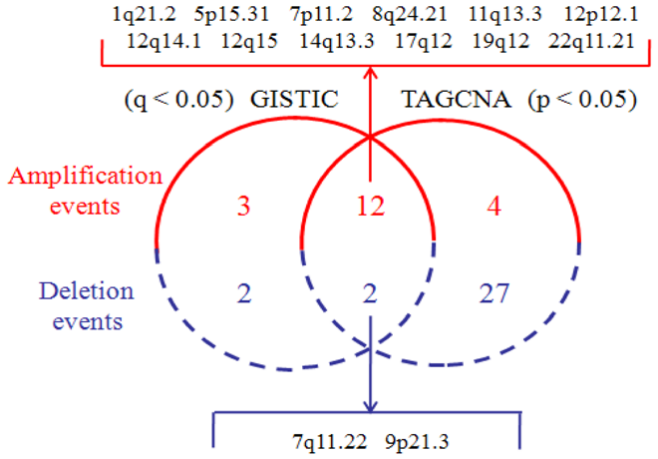
A Venn diagram comparison between GISTIC and TAGCNA in terms of SCEs identified in the lung adenocarcinoma data. The overlapped amplification and deletion events are listed in the top and bottom of the Venn diagram. Here, we use the common cutoffs q<0.05 and p<0.05 for GISTIC and TAGCNA, respectively.

We use Venn diagram to compare SCEs resulted from TAGCNA with that from GISTIC in Figure 5. TAGCNA provides statistical support for 80% of the amplification events and 50% of the deletion events that GISTIC detected. Most of the overlapped SCEs encompass one or more oncogenes or tumor suppressor genes. In addition, a part of the non-overlapped deletion SCEs of TAGCNA is supported by CMDS result [13] such as 10q21.2 and 15q11.1. Furthermore, we suppose that existing approaches might miss some SCEs shown to be statistical and biological significance. Here we characterize one SCE (21q22.2) uniquely identified by TAGCNA. Deletion at 21q22.2 (p-value <0.001) occurs in 11 (3%) subjects with copy number below 1.2 and occurs in 24 (6.5%) subjects with copy number below 1.5, and the minimum inferred copy number is 0.3. This SCE covers three genes (PCP4, DSCAM, and TMPRSS3), in which TMPRSS3 has been validated to be clinically and biologically associated with human diseases [21], [22].

In Figure 5, it is easy to note that the number of new SCEs detected by TAGCNA in deletion is larger than that in amplification. Examination of the copy number profiles in the lung adenocarcinoma dataset and the detected SCEs reveals two reasons for this discrepancy. The most common explanation is that the deletion event is present more frequently than amplification event [19] and most of the deletions are heterogeneity (i.e. loss of one copy) [3], such as seen in the lung adenocarcinoma samples for 17p11.2 deletion. Here, 6.8% of the samples exhibit deletion magnitude between 1 and 1.5, while only a few (1%) of the samples exhibit deletion magnitude below 1. Accordingly, 17p11.2 is a less-extreme region (i.e. frequency and magnitude are relatively low), which may not be discovered under the null distribution contributed by multiple large deletion SCEs. However, such regions would reach significance by removing SCEs from the genome and re-creating new null distributions performed by TAGCNA. The second explanation is that the correlation coefficient among the deletion probes in this particular dataset is relatively higher than that among the amplification probes, thus the detection of individual probes without considering correlations would lead to a higher conservativeness. For example, the deletion at 7q11.22 is assigned p-value less than 0.001 by TAGCNA, but it is reported by GISTIC with q-value more than 0.025.

#### Result on the prostate adenocarcinoma dataset

**Figure 6:**
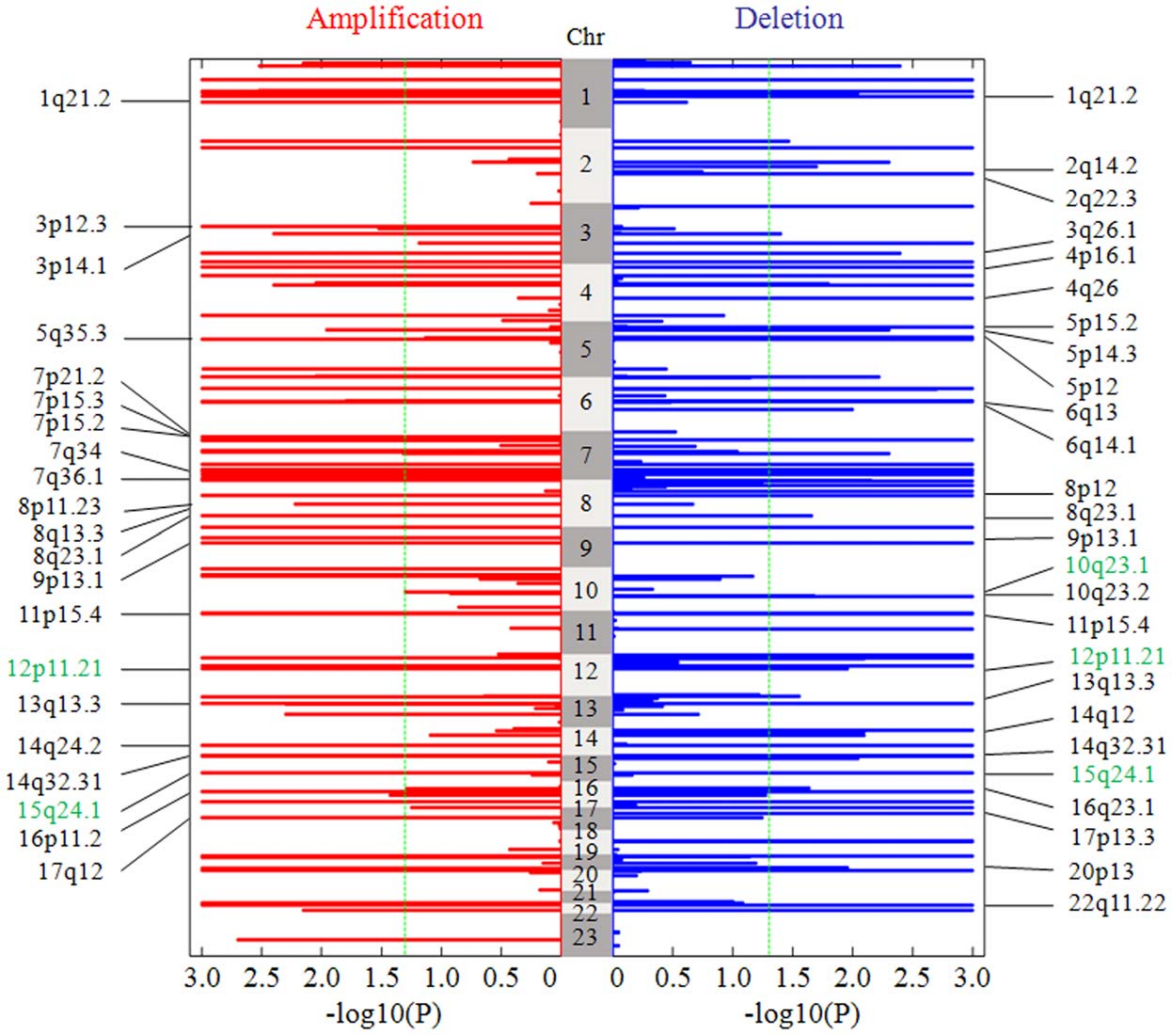
The landscape of statistical significance levels of the genome in the 82 prostate adenocarcinoma subjects. −log10 (p-values) are given for amplification and deletion regions respectively. The dashed green line is placed at 1.3 (corresponding p-value of 0.05) as a cutoff for calling significant consensus events. Chromosome 23 indicates the sex chromosome. Many important SCEs are listed in the both sides of the figure.

The significance landscape of the whole genome analyzed by TAGCNA on the prostate adenocarcinoma dataset is given in Figure 6. A total of 91 amplification SCEs and 97 deletion SCEs are identified in the dataset, and the covered genes are listed in Table S2. Most of these SCEs are shown to be biologically relevant and are supported by previously reported results. For example, amplifications at 1q21.1, 7p21.2, 7q36.1, 8q13.3, 8q23.1, 9p13.1, 14q24.2, 14q32.31, and 16p11.2 are introduced by Outi [23], where 7p21.2 contains transcription factor ETV1, which was found to be substantially over-expressed in a subset of prostate cancers, and 14q24.2 is closely adjacent to HIF1A, the protein encoded by this gene has been shown to be over-expressed in many prostate cancers; and amplifications at 11p15.4, 3p12.3, 3p12.1, 13q13.3, 17q12, 7p15.3, 7p15.2, 7q34, 5q35.3, and 8p11.23 are reported by other authors [24], [25], [26], [27]. Deletions at 2q14.2, 4p16.1,4q26, 6q13, 9p13.1, 10q23.2, 16q23.1, and 17p13.3 are introduced by Outi [23], where 10q23.2 and16q23.1 are extremely close to important potential tumor suppressor genes PTEN and HSD17B2; and deletions at 8p12, 1q21.2, 5p15.2,5p14.3,5p12,14q12, 14q32.31, 6q14.1,13q13.3, 3q26.1, 11p15.4, and 20p13 are presented by other authors [25], [26], [27], [28]. These results indicate that TAGCNA is applicable to the analysis of real CNA datasets.

Moreover, many additional amplification and deletion SCEs are identified by TAGCNA (A part of them are listed in Table 2), which can be used for further investigation. For instance, 12p11.21 and 15q24.1 encompass genes FGD4 and HCN4 respectively. Mutations in these genes have been associated with Charcot Marie Tooth disease type 4H [29] and sick sinus syndrome2 [30] respectively. We note that the two SCEs show statistical significance (p-value <0.001) in both amplification and deletion situations. Another deletion SCE 10q23.1 contains GRID1, which has been shown to be related with the increased risk of developing schizophrenia [31].

**Table 2:** A part of additional SCEs identified by TAGCNA in prostate adenocarcinoma dataset.

		Amplification				Deletion			
SCE	Candidate gene	p-value	>3.6	>2.5	Max.CN	p-value	<1.2	<1.5	Min.CN
12p11.21	FGD4	<0.001	19 (23.2)	34 (40.5)	6.3	<0.001	7 (8.54)	16 (19.5)	0.24
15q24.1	HCN4	<0.001	17 (20.7)	38 (46.3)	5.3	<0.001	15 (18.3)	17 (20.7)	0.37
10q23.1	GRID1	1.00				0.01	9 (10.9)	18 (21.9)	0.47

">3.6" means the number of copies of subjects at the SCE location larger than 3.6, and the same to ">2.5"; "<1.2" means the number of copies less than 1.2, and the same to "<1.5";

G(K), *e.g.* 19(23.2), denotes the number of subjects (percentage) above the threshold;

Max.CN/Min.CN represents the maximum or minimum inferred copy number.

## Discussion

### General Summary

Identification of SCEs in somatic copy number data has proven to be an effective technique to discover cancer driver genes. In this article we propose a novel approach TAGCNA, aiming to increase the statistical power for detecting SCEs. TAGCNA is motivated by carefully considering biological and statistical significance. To preserve the inherent correlations in CNA data and to make a consistency between statistic and permutation procedure, TAGCNA constructs CNA blocks and tests the statistical significance of tag markers that represent the blocks. To correct p-values assigned to tag markers, TAGCNA adopts a peel-off permutation scheme to generate a reasonable null distribution.

We perform simulation studies to examine performance of TAGCNA in comparison with that of the CMDS method. Since both of the methods have considered the correlations among adjacent markers and have modeled the average correlations using a window size, for a fair comparison, we choose *w*  = 20, as the default value of the CMDS algorithm [13], in the simulation studies. The result shows that TAGCNA presents higher true positive rate at the same false positive rate in various simulation datasets than that of the CMDS method. The most common explanation is that CMDS measures CNAs only using the correlations, which usually exist in both SCEs and background CNAs. Especially when the background CNAs are very common, the power of identifying SCEs using correlation score would be decreased. We further test the type I error rate of TAGCNA using simulated null datasets. The result indicates that TAGCNA performs well and is slightly conservative compared to the p-value threshold of 0.05. In application to two real CNA datasets, TAGCNA readily identifies SCEs that are known to be involved with cancer driver genes and provides new SCEs with potential biological relevance. TAGCNA is an extremely flexible approach. Specifically, it is suitable for analyzing CNA data profiled from any array platform such as Affymetrix Human Mapping 250K STY SNP Array and the Affymetrix Genome-Wide Human SNP Array 6.0. For the adjustment for multiple comparisons, TAGCNA is similar to existing methods such as STAC [5], MSA [32], and DiNAMIC [9], using the max-T procedure to control the family-wise error rate (FWER).

As for the algorithm parameter selection of *w* in real applications, there is no general guideline about how to determine its value since different kinds of cancers usually have different rates and magnitudes of CNAs [6], which would lead to various degrees of correlations among markers and various lengths of CNA blocks. We have tested this empirically in a large number of experiments using different window sizes and have found that a value of *w* taking between 10 and 50 would be a suitable choice in most contexts. Generally, relatively lower values are helpful to identify focal CNAs while relatively higher values are helpful to identify broad CNA regions (the size of them is near that of a chromosome arm). Since our main objective here is to identify focal CNAs, we adopt a relatively lower value of *w* (i.e. *w*  = 20) in the implementations of TAGCNA on the two real datasets.

TAGCNA can be performed on either individual chromosomes or genome-wide. Since different chromosomes may have different background CNAs and perform different roles in human diseases, we implement TAGCNA by permutations on individual chromosomes in the analysis of lung and prostate adneocarcinoma cancers in this article. In general, focal SCEs might be identified more easily than broad SCEs in this scheme due to the limited length of individual chromosomes, while broad SCEs might be likely identified on genome-wide permutation and they are usually regarded to contribute important biological consequences to cancers [1], [3], [8]. To mirror this, we have also performed TAGCNA on genome-wide permutation and have obtained a larger number of broad SCEs (data not shown here). An additional factor to affect the identification of focal and broad SCEs is the threshold definitions of amplification and deletion. Higher thresholds might be focused on focal SCEs while lower threshold might be focused on broad SCEs [8]. As aforementioned, our purpose here is to identify focal SCEs, we choose relatively higher thresholds for calling amplification (greater than 3.6 copies) and deletion (less than 1.2 copies). Moreover, we also focus on identifying chromosome-specific SCEs, and choose a commonly used p-value threshold of 0.05 [5], [9], [13] to determine significant in real applications. From the viewpoint of whole genome, it is certainly true that the p-value threshold is too liberal since there are 23 multiple tests in either amplification or deletion analysis. In this case, the threshold of 0.05/23 seems to be more reasonable. However, from the viewpoint of individual chromosomes, the scaled threshold might be too conservative and would omit some chromosome-specific SCEs [5].

### The Impact of Contamination of Normal Cell on the Detection Power

**Figure 7:**
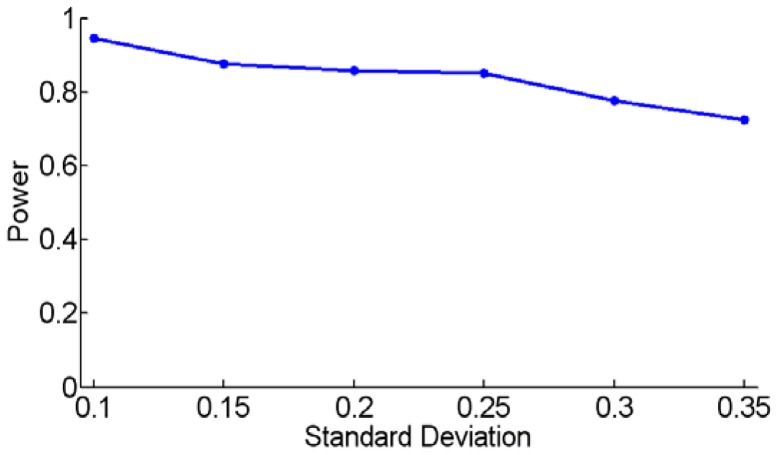
Power curve for TAGCNA for simulated datasets containing two SCEs. The normal tissue contamination in the datasets is generated under a Gaussian distribution with mean 0.6 and varying standard deviation from 0.1 to 0.35. The power is averaged over 100 simulation replications.

Since tissue samples often consist of a mixture of cancer and normal cells, the produced somatic CNA profiles are the weighted sum of copy numbers contributed by cancer and normal cells [33]. Accordingly, the measured copy numbers are smaller (or larger) than the true values in amplification events (or deletion events). It is generally regarded that the fraction of the normal cells contained in the tissue samples may affect the power of calling amplifications and deletions, as well as affect the detection power of significant consensus CNA events [33]. To investigate the impact of the normal cell fraction on the power of TAGCNA for identifying SCEs, we simulate various datasets from a mixture of cancer and normal cells and implement TAGCNA on these datasets. The fraction of normal cells is generated from a Gaussian distribution with mean 0.6 and varying standard deviation from 0.1 to 0.35. In each dataset we insert one amplified and one deleted ground truth SCEs with frequency of 0.15. We use a significance cutoff 0.05 to determine if TAGCNA identifies the SCEs, and count the power based on 100 replications simulated under each kind of the Gaussian distributions. Figure 7 shows the power curve, indicating that the power to identify SCEs decreases gradually with the increased standard deviation. However, one possible strategy to deal with this issue is to recover the true copy number profiles of cancer tissues by estimating the fraction of normal cells. Currently there are a couple of studies have attempted this approach [33], [34].

### Future Work

Four directions for future research to extend TAGCNA are envisioned. The first is to enhance the power of TAGCNA by combining normal cell contamination correction method such as BACOM (Bayesian Analysis of COpy number Mixtures) [33]. The copy number corrected samples may not only make the definition of amplification or deletion more accurate, but also make the null distribution more reasonable since it often incorporates the amplitude of CNA signals. The second direction is to explore a new way to select tag markers using an independent set of normal individuals with similar genetic background as the samples analyzed. This will be helpful to avoid bias in the determination of background CNA blocks and thus to improve the power of detecting significant CNAs. The third direction is to incorporate gene expression data in the analysis of cancer copy number data. The identified SCEs from CNA data can be associated with the RNA expression levels in cancer samples to explore functional consequences. This is an intuitive extension aimed to validate the biological and clinical relevance of SCEs. The abundant and high-quality data sets with clinical information published by the TCGA project (http://cancergenome.nih.gov/) would facilitate these studies. The last extension is to apply TAGCNA to analyze CNV (copy number variation) data from normal populations and LOH (loss of heterozygosity) data in cancer samples. These data are of course different from CNAs in terms of density, amplitude, etc. TAGCNA would provide statistical strictness to this analysis and may reveal potential copy number change patterns associated with phenotypes.

### Conclusions

In conclusion, TAGCNA can be used to identify SCEs from random background CNAs in various cancer genomes, and may obtain an acceptable type I error rate. Its permutation scheme on tag CNA markers and its peel-off procedure in generating null distribution greatly contributes to this outcome. TAGCNA is very flexible in performing permutation within chromosome or across whole genome. Users do not need to do any extra work to choose either type of permutations they want to implement.

The experiment results suggest that TAGCNA is an improved algorithm to achieve better sensitivity while maintaining the same specificity and can provide important gene candidates for studying tumor development and progression. The TAGCNA algorithm is implemented in R, and has been examined on Windows and Linux platforms.

## Supporting Information

Table S1 Genes covered by the SCEs identified in the lung adenocarcinoma dataset.
(DOC)

Table S2 Genes covered by the SCEs identified in the prostate adenocarcinoma dataset.
(DOC)
