# Supplementary material for: TAGCNA: A Method to Identify Significant Consensus Events of Copy Number Alterations in Cancer
Source: PLoS One. 2012 Jul 18;7(7):e41082. doi: 10.1371/journal.pone.0041082 (PMC3399811; doi:10.1371/journal.pone.0041082)
Supplement: Table S1 — Genes covered by the SCEs identified in the lung adenocarcinoma dataset. (DOC) [file pone.0041082.s001.doc]

**Table S1.** Details about the implicated SCEs and full list of genes covered by these SCEs, derived from the lung adenocarcinoma dataset. (Chr#: chromosome index, Start: Start point of SCEs, End: End point of SCEs.) (Based on biomaRt at http://bioconductor.org/biocLite.R)

| **Chr#** | **SCE** | | **Cyto-**  **band** | **Genes covered by the SCE** |
| --- | --- | --- | --- | --- |
|  | **Start** | **End** |  |  |
| ***Amplification*** | | | | |
| 1 | 77036886 | 77386105 | 1p31.1 | ST6GALNAC3,ST6GALNAC5,TPI1P1 |
| 1 | 147483025 | 147794693 | 1q21.2 | RNU1-6,NBPF24,FAM108A2 |
| 1 | 212314333 | 212505967 | 1q32.3 | PPP2R5A |
| 5 | 9641308 | 9810645 | 5p15.31 |  |
| 7 | 54325519 | 55947837 | 7p11.2 | VSTM2A,SEC61G,EGFR,LANCL2,VOPP1,SEPT14,CALM1P2,FKBP9L,SUMO2P3,CICP11,CICP12 |
| 8 | 128492832 | 129776279 | 8q24.21 | MIR1208,MIR1204,MIR1205,MIR1207,MYC,TMEM75,PVT1 |
| 11 | 68438569 | 70057390 | 11q13.3 | ANO1,FADD,FGF3,FGF4,MRGPRD,MRGPRF,TPCN2,MYEOV,CCND1,ORAOV1,FGF19,IGHMBP2,GAL,MTL5,CPT1A,MRPL21,ANO1-AS2,ANO1-AS1 |
| 12 | 24839309 | 25860141 | 12p12.1 | CASC1,LRMP,C12orf77,IFLTD1,BCAT1,LYRM5,KRAS |
| 12 | 56231445 | 56679770 | 12q14.1 | ANKRD52,COQ10A,CS,RNF41,OBFC2B,SLC39A5,CDK2,RAB5B,SUOX,IKZF4,RPS26,ERBB3,PA2G4,RPL41,ZC3H10,ESYT1,MYL6B,MYL6,SMARCC2,MMP19,WIBG,DGKA,PMEL,GSTP1P1 |
| 12 | 66288294 | 69352739 | 12q15 | SNORA70G,HMGA2,LLPH,TMBIM4,IRAK3,HELB,GRIP1,CAND1,DYRK2,IFNG,IL26,IL22,MDM1,RAP1B,NUP107,SLC35E3,MDM2,CPM,RBMS1P1,KRT8P22 |
| 14 | 32824020 | 38284539 | 14q13.3 | RNU1-7,RNU1-8,RNU7-41P,AKAP6,NPAS3,EGLN3,SPTSSA,EAPP,SNX6,CFL2,BAZ1A,SRP54,FAM177A1,PPP2R3C,KIAA0391,PSMA6,NFKBIA,INSM2,RALGAPA1,BRMS1L,MBIP,SFTA3,NKX2-1,NKX2-8,PAX9,SLC25A21,MIPOL1,FOXA1,TTC6,RPS19P3,NKX2-1-AS1,PHKBP2,RPL29P3 |
| 15 | 91889094 | 92056428 | 15q26.1 |  |
| 17 | 10739738 | 10868323 | 17p12 | PIRT |
| 17 | 34801121 | 35087126 | 17q12 | TBC1D3H,TBC1D3G,ZNHIT3,MYO19,PIGW,GGNBP2,DHRS11,MRM1 |
| 19 | 34283030 | 35545153 | 19q12 | ZNF807,KCTD15,RPS4XP21,LSM14A,KIAA0355,GPI,PDCD2L,UBA2,WTIP,SCGBL,ZNF302,ZNF181,ZNF599,ZNF30,ZNF792,GRAMD1A,SCN1B,HPN |
| 22 | 19166938 | 19768928 | 22q11.21 | CLDN5,SEPT5,GP1BB,TBX1,CLTCL1,HIRA,C22orf39,MRPL40,UFD1L,CDC45 |
| ***Deletion*** | | | | |
| 1 | 162336261 | 162579147 | 1q23.3 | NOS1AP,,C1orf226,C1orf111,SH2D1B,UHMK1,UAP1,UQCRBP2 |
| 2 | 14622596 | 14763089 | 2p24.3 |  |
| 3 | 31403429 | 31696708 | 3p23 | STT3B,THRAP3P1 |
| 3 | 52830905 | 53118941 | 3p21.1 | ITIH3,ITIH4,MUSTN1,TMEM110-MUSTN1,TMEM110,SFMBT1,ITIH4-AS1 |
| 4 | 9932206 | 9966143 | 4p16.1 | SLC2A9 |
| 4 | 42803921 | 43173271 | 4p13 | GRXCR1 |
| 5 | 3667352 | 3796669 | 5p15.33 |  |
| 6 | 29941927 | 30026233 | 6p22.1 | HCG9,DDX39BP2,MCCD1P2,ZNRD1-AS1,HCG4P3,HLA-J,ETF1P1 |
| 7 | 69694776 | 69815120 | 7q11.22 | AUTS2 |
| 8 | 1262791 | 1446639 | 8p23.3 |  |
| 8 | 5503023 | 5672160 | 8p23.2 |  |
| 8 | 128178958 | 128466129 | 8q24.21 | POU5F1B |
| 9 | 21068375 | 24409824 | 9p21.3 | MIR31,IFNB1,IFNW1,IFNA21,IFNA4,IFNA7,IFNA10,IFNA16,IFNA17,IFNA14,IFNA5,KLHL9,IFNA6,IFNA13,IFNA2,IFNA8,IFNA1,IFNE,MTAP,C9orf53,CDKN2A,IFNWP4,IFNWP15,IFNWP9,IFNWP18,IFNWP5,IFNA22P,IFNA20P,IFNA11P,IFNA12P,IFNWP2,IFNWP19,KHSRPP1,CDKN2B-AS1,UBA52P6,SUMO2P2,CDKN2B,DMRTA1,ELAVL2 |
| 10 | 62103446 | 62266068 | 10q21.2 | ANK3 |
| 11 | 4124008 | 4389782 | 11p15.4 | RRM1,OR52B4,,OR55B1P |
| 11 | 12239484 | 12337523 | 11p15.3 | MICAL2,MICALCL |
| 12 | 82651391 | 83257551 | 12q21.31 | CCDC59,C12orf26,TMTC2 |
| 13 | 23241675 | 23495985 | 13q12.12 | FTH1P7,DDX39AP1,RPL7AP73,IPMKP1,RFESDP1,BASP1P1,NUS1P2 |
| 13 | 102123612 | 102379142 | 13q33.1 | ITGBL1,FGF14 |
| 15 | 19922588 | 20588197 | 15q11.1 | HERC2P3 |
| 17 | 16349340 | 16827203 | 17p11.2 | KRT17P1,CCDC144A,ZNF624,NCRNA00188,UPF3AP1,KRT16P2,TBC1D27,C17orf76,ZNF287 |
| 17 | 51510249 | 51556430 | 17q22 |  |
| 17 | 74682195 | 74830587 | 7q25.1 | MIR636,MXRA7,JMJD6,METTL23,SRSF2,MFSD11  18,55282774,55370142,18q21.31,NARS,ATP8B1 |
| 18 | 61899757 | 62259034 | 18q22.1 |  |
| 19 | 48063702 | 48704720 | 19q13.33 | SNORD23,,RPL23AP80,TPRX2P,GLTSCR1,EHD2,GLTSCR2,TPRX1,CRX,SULT2A1,BSPH1,ELSPBP1,CABP5,PLA2G4C,LIG1,C19orf68 |
| 19 | 53564869 | 54056323 | 19q13.42 | VN1R6P,,ZNF761,TPM3P6,ZNF160,ZNF415,ZNF347,ZNF665,ZNF677,VN1R2,VN1R4,BIRC8,ZNF845,ZNF525,ZNF765,ZNF813,ZNF331 |
| 20 | 54992798 | 55077644 | 20q13.31 | CASS4,C20orf43,GCNT7,RPL39P |
| 21 | 41203234 | 41401386 | 21q22.2 | PCP4,DSCAM,TMPRSS3 |
