# Supplementary material for: TAGCNA: A Method to Identify Significant Consensus Events of Copy Number Alterations in Cancer
Source: PLoS One. 2012 Jul 18;7(7):e41082. doi: 10.1371/journal.pone.0041082 (PMC3399811; doi:10.1371/journal.pone.0041082)
Supplement: Table S2 — Genes covered by the SCEs identified in the prostate adenocarcinoma dataset. (DOC) [file pone.0041082.s002.doc]

**Table S2.** Details about the implicated SCEs and full list of genes covered by these SCEs, derived from the prostate adenocarcinoma dataset. (Chr#: chromosome index, Start: Start point of SCEs, End: End point of SCEs.) (Based on biomaRt at http://bioconductor.org/biocLite.R)

| **Chr#** | **SCE** | | **Cyto-**  **band** | **Genes covered by the SCE** |
| --- | --- | --- | --- | --- |
|  | **Start** | **End** |  |  |
| ***Amplification*** | | | | |
| 1 | 15701329 | 15733149 | p36.21 | FHAD1 |
| 1 | 25088617 | 25108701 | p36.11 | CLIC4 |
| 1 | 71418471 | 71506681 | p31.1 | PTGER3 |
| 1 | 104110702 | 104152171 | p21.1 | ACTG1P4,AMY2B |
| 1 | 108241606 | 108250254 | p13.3 | VAV3 |
| 1 | 111325204 | 111358788 | p13.3 |  |
| 1 | 148763480 | 148897492 | q21.2 | NKAIN1P1 |
| 1 | 149218429 | 149261859 | q21.2 | RNU1-5 |
| 1 | 165808424 | 165823534 | q24.1 | UCK2 |
| 2 | 34094127 | 34144863 | p22.3 |  |
| 2 | 51689956 | 51718534 | p16.3 |  |
| 3 | 67762036 | 67792426 | p14.1 |  |
| 3 | 74348287 | 74374798 | p12.3 | CNTN3 |
| 3 | 99033538 | 99051042 | q12.1 |  |
| 3 | 161996213 | 162038648 | q26.1 |  |
| 3 | 192175685 | 192241616 | q28 | FGF12,FGF12-AS2,FGF12-AS3 |
| 4 | 9733184 | 9753556 | p16.1 |  |
| 4 | 9868325 | 9879397 | p16.1 | SLC2A9 |
| 4 | 33882894 | 33989696 | p15.1 |  |
| 4 | 62341381 | 62348315 | q13.1 | LPHN3 |
| 4 | 63385977 | 63422090 | q13.1 |  |
| 4 | 67914168 | 67925379 | q13.2 |  |
| 4 | 170394108 | 170422606 | q33 | NEK1 |
| 4 | 171164768 | 171171844 | q33 |  |
| 5 | 21049012 | 21083743 | p14.3 |  |
| 5 | 56707210 | 56746721 | q11.2 | SALL4P1 |
| 5 | 153598448 | 153622862 | q33.2 | MFAP3,GALNT10 |
| 5 | 174354979 | 174376106 | q35.2 |  |
| 5 | 177870493 | 177897394 | q35.3 | COL23A1 |
| 6 | 30787606 | 30853432 | p21.33 | NCRNA00243,TIGD1L,DDR1 |
| 6 | 31568198 | 31619328 | p21.33 | SNORA38,AIF1,PRRC2A,BAG6,UQCRHP1 |
| 6 | 31711569 | 31719818 | p21.33 | MSH5-C6orf26,MSH5 |
| 6 | 73542130 | 73564745 | q13 | KCNQ5 |
| 6 | 76269766 | 76335739 | q14.1 | SENP6 |
| 6 | 77883591 | 78094736 | q14.1 |  |
| 7 | 15153296 | 15180823 | p21.2 |  |
| 7 | 22125376 | 22138106 | p15.3 |  |
| 7 | 25698549 | 25728993 | p15.2 |  |
| 7 | 53624162 | 53637706 | p12.1 |  |
| 7 | 63137056 | 63156789 | q11.21 |  |
| 7 | 69101397 | 69121503 | q11.22 | AUTS2 |
| 7 | 107951719 | 107964211 | q31.1 | NRCAM |
| 7 | 124374072 | 124446591 | q31.33 | GPR37 |
| 7 | 131840228 | 131891080 | q32.3 | PLXNA4 |
| 7 | 140175568 | 140207290 | q34 | MKRN1 |
| 7 | 140700443 | 140743853 | q34 | MRPS33 |
| 7 | 152406888 | 152412200 | q36.1 |  |
| 8 | 572635 | 596355 | p23.3 | ERICH1 |
| 8 | 38150501 | 38235866 | p11.23 | WHSC1L1 |
| 8 | 70839882 | 70855548 | q13.3 |  |
| 8 | 110360648 | 110368935 | q23.1 | Naz |
| 8 | 110401142 | 110434701 | q23.1 | PKHD1L1 |
| 9 | 587273 | 592903 | p24.3 | KANK1 |
| 9 | 22850885 | 22897647 | p21.3 |  |
| 9 | 38600329 | 38632301 | p13.1 | ANKRD18A,FAM201A |
| 9 | 38986237 | 39079270 | p13.1 | VN2R3P,CNTNAP3 |
| 10 | 4202542 | 4224142 | p15.1 |  |
| 10 | 17501181 | 17508054 | p12.33 |  |
| 10 | 24042322 | 24069122 | p12.2 | KIAA1217 |
| 11 | 4891601 | 4906090 | p15.4 | MMP26,OR51T1,OR51H2P |
| 11 | 5429310 | 5439273 | p15.4 | HBG2,HBE1 |
| 12 | 9318132 | 9345815 | p13.31 | PZP |
| 12 | 32688889 | 32717601 | p11.21 | FGD4 |
| 12 | 43484124 | 43517290 | q12 |  |
| 13 | 18646349 | 18679766 | q11 |  |
| 13 | 19005736 | 19041738 | q11 | ZNF962P |
| 13 | 36728965 | 36763144 | q13.3 | SOHLH2,CCDC169-SOHLH2 |
| 13 | 37637248 | 37646574 | q13.3 |  |
| 13 | 70618077 | 70625773 | q21.33 | KLHL1 |
| 14 | 72194938 | 72245034 | q24.2 | SIPA1L1 |
| 14 | 103131223 | 103175166 | q32.31 | RCOR1 |
| 15 | 18838066 | 18846971 | p11.1 |  |
| 15 | 73586546 | 73688021 | q24.1 | NEO1,HCN4 |
| 16 | 31305300 | 31338487 | p11.2 | ITGAM |
| 16 | 53868794 | 53871039 | q12.2 | FTO |
| 16 | 76271662 | 76299582 | q23.1 |  |
| 17 | 36225832 | 36253313 | q12 |  |
| 19 | 5354382 | 5409229 | p13.3 |  |
| 19 | 12377324 | 12394259 | p13.2 | ZNF44 |
| 20 | 1484359 | 1520451 | p13 | SIRPD |
| 22 | 15849757 | 15880035 | q11.1 |  |
| 22 | 17881687 | 17926995 | q11.21 | CECR2,,CLCP1 |
| 22 | 19164255 | 19229146 | q11.21 | CLTCL1,SLC25A1 |
| 22 | 20751976 | 20762532 | q11.21 | ZNF74 |
| 22 | 22476323 | 22528577 | q11.22 | IGLV4-60,,SOCS2P2 |
| 22 | 37038809 | 37049907 | q12.3 | CACNG2 |
| 23 | 97211167 | 97219623 | q12.3 |  |
| ***Deletion*** | | | | |
| 1 | 25088751 | 25112627 | p36.11 | CLIC4 |
| 1 | 71407508 | 71486987 | p31.1 | PTGER3 |
| 1 | 108227128 | 108250254 | p13.3 | VAV3 |
| 1 | 111264575 | 111369154 | p13.3 |  |
| 1 | 148832747 | 148885584 | q21.2 | NKAIN1P1 |
| 1 | 149233371 | 149257536 | q21.2 |  |
| 2 | 35300746 | 35344190 | p22.3 |  |
| 2 | 51691732 | 51724006 | p16.3 |  |
| 2 | 105158936 | 105168708 | q12.1 |  |
| 2 | 121472182 | 121502382 | q14.2 | GLI2 |
| 2 | 144318653 | 144371999 | q22.3 | ARHGAP15 |
| 3 | 6481425 | 6511329 | p26.1 |  |
| 3 | 99024544 | 99088778 | q12.1 |  |
| 3 | 129177631 | 129237921 | q21.3-q22.1 | IFT122 |
| 3 | 162013324 | 162020506 | q26.1 |  |
| 3 | 192205300 | 192225890 | q28 | FGF12 |
| 4 | 9868325 | 9877440 | p16.1 | SLC2A9 |
| 4 | 33884935 | 33972257 | q15.1 |  |
| 4 | 63322293 | 63422138 | q13.1 | HMGN1P11 |
| 4 | 67944856 | 68081357 | q13.2 |  |
| 4 | 114169495 | 114187390 | q26 | ANK2 |
| 5 | 12491420 | 12526787 | p15.2 |  |
| 5 | 21048388 | 21061372 | p14.3 |  |
| 5 | 45060035 | 45092020 | p12 |  |
| 5 | 56706676 | 56746721 | q11.2 | SALL4P1 |
| 5 | 177873237 | 177893037 | q35.3 | COL23A1 |
| 6 | 30800517 | 30834917 | p21.33 | TIGD1L |
| 6 | 31563930 | 31588250 | p21.33 | AIF1,UQCRHP1 |
| 6 | 33508546 | 33521947 | p21.31 |  |
| 6 | 73548748 | 73564577 | q13 | KCNQ5 |
| 6 | 76293840 | 76351684 | q14.1 | SENP6 |
| 6 | 102301424 | 102359519 | q16.3 | GRIK2 |
| 7 | 22128178 | 22136503 | p15.3 |  |
| 7 | 69101420 | 69124899 | q11.22 | AUTS2 |
| 7 | 107951695 | 107960358 | q31.1 | NRCAM |
| 7 | 124424341 | 124426841 | q31.33 |  |
| 7 | 131842266 | 131871638 | q32.3 | PLXNA4 |
| 7 | 139874586 | 139885971 | q34 | JHDM1D |
| 7 | 140173500 | 140207290 | q34 | MKRN1 |
| 7 | 140721667 | 140735294 | q34 |  |
| 8 | 577539 | 585908 | p23.3 | ERICH1 |
| 8 | 2647613 | 2658916 | p23.2 |  |
| 8 | 11516588 | 11519658 | p23.1 |  |
| 8 | 11535557 | 11536950 | p23.1 | GATA4 |
| 8 | 24413355 | 24425441 | p21.1 |  |
| 8 | 38139434 | 38253312 | p11.23 | WHSC1L1,LETM2 |
| 8 | 110397324 | 110415463 | q23.1 | PKHD1L1 |
| 9 | 562920 | 582537 | p24.3 |  |
| 9 | 38593329 | 38644028 | p13.1 | ANKRD18A,FAM201A |
| 10 | 87849967 | 87866225 | q23.1 | GRID1 |
| 10 | 88134090 | 89107280 | q23.2 | RNU1-19P,WAPAL,OPN4,LDB3,BMPR1A,MMRN2,SNCG,C10orf116,FAM25A,GLUD1,FAM35A,FAM22A,RPL7AP8,AGAP11,BMS1P3 |
| 11 | 3349918 | 3364104 | p15.4 | ZNF195 |
| 11 | 4881742 | 4903055 | p15.4 | MMP26,OR51H1P,OR51T1,OR51H2P |
| 11 | 5429310 | 5439273 | p15.4 | HBG2,HBE1 |
| 11 | 5665603 | 5696742 | p15.4 | HBG2,TRIM34,TRIM6-TRIM34,TRIM5 |
| 11 | 50164142 | 50218697 | p11.12 |  |
| 12 | 692230 | 743765 | p13.33 | NINJ2 |
| 12 | 9311883 | 9318132 | p13.31 | PZP |
| 12 | 10880587 | 10943344 | p13.2 |  |
| 12 | 32688889 | 32717601 | p11.21 | FGD4 |
| 12 | 43484124 | 43529276 | q12 |  |
| 12 | 129266681 | 129291485 | q24.32 | SLC15A4 |
| 13 | 36704033 | 36764076 | q13.3 | DCLK1,SOHLH2,CCDC169-SOHLH2 |
| 14 | 24684000 | 24701751 | q12 | MDP1,NEDD8-MDP1,NEDD8,GMPR2,CHMP4A |
| 14 | 28577042 | 28604670 | q12 |  |
| 14 | 40444388 | 40455379 | q21.1 |  |
| 14 | 72207941 | 72234174 | q24.2 | SIPA1L1 |
| 14 | 103127029 | 103195770 | q32.31 | RCOR1 |
| 14 | 103369840 | 103443297 | q32.32 | TRAF3,AMN,CDC42BPB |
| 14 | 103473639 | 103570179 | q32.32 | RPL13P6,CDC42BPB,EXOC3L4 |
| 15 | 18841578 | 18846375 | p11.1 |  |
| 15 | 19123078 | 19159195 | q11.1 |  |
| 15 | 28020137 | 28038190 | q12 | OCA2 |
| 15 | 73612391 | 73675902 | q24.1 | HCN4 |
| 16 | 19392231 | 19413313 | p12.3 |  |
| 16 | 31305656 | 31352250 | p11.2 | ITGAM |
| 16 | 76271705 | 76290822 | q23.1 |  |
| 17 | 611794 | 660922 | p13.3 | VPS53,FAM57A,GEMIN4,,DBIL5P |
| 17 | 17957824 | 18010015 | p11.2 | C17orf39,DRG2 |
| 18 | 36163380 | 36190372 | q12.2 |  |
| 18 | 37828795 | 37843120 | q12.3 |  |
| 19 | 5355312 | 5406385 | p13.3 |  |
| 19 | 7113395 | 7133962 | p13.2 | INSR |
| 19 | 56306272 | 56343302 | q13.43 | NLRP11 |
| 20 | 1494811 | 1519903 | p13 | SIRPD |
| 22 | 23133922 | 23176390 | q11.22 | MIR650,IGLV2-11,IGLV3-10,IGLV3-9,IGLV2-8 |
| 22 | 37021829 | 37050745 | q12.3 | CACNG2 |
